# Perceived Stigma of Patients Undergoing Treatment with Cannabis-Based Medicinal Products

**DOI:** 10.3390/ijerph19127499

**Published:** 2022-06-19

**Authors:** Lucy J. Troup, Simon Erridge, Beata Ciesluk, Mikael H. Sodergren

**Affiliations:** 1 Division of Psychology, School of Education and Social Sciences, University of the West of Scotland, Paisley PA1 2BE, UK; b00307252@studentmail.uws.ac.uk; 2 Medical Cannabis Research Group, Imperial College London, London SW7 2BX, UK; simon.erridge12@imperial.ac.uk (S.E.); m.sodergren@imperial.ac.uk (M.H.S.); 3 Sapphire Medical Clinics, London W1G 9PF, UK

**Keywords:** cannabis, medical cannabis, cannabinoids, social stigma, health services accessibility

## Abstract

Cannabis-based medicinal products (CBMPs) are prescribed with increasing frequency. This study aimed to investigate the perceived stigma attached to patients prescribed CBMPs in the UK to establish its prevalence. A qualitative survey was developed by an expert multidisciplinary group and data were collected via Qualtrics. In total, 2319 patients on CBMP therapy were invited to take part in this study. 450 (19.4%) participants completed the questionnaire. In total, 81.3% (*n* = 366), 76.9% (*n* = 346), and 61.3% (*n* = 276) of participants reported feeling very comfortable or comfortable telling friends, family, and medical professionals, respectively, about their treatment. Participants thought that friends (*n* = 372; 82.7%) and family (*n* = 339; 75.3%) were very approving or somewhat approving of their CBMP prescription. However, participants thought that only 37.8% (*n* = 170) of healthcare professionals and 32.9% (*n* = 148) of society in general were very approving or somewhat approving of their CBMP prescription. 57.1% (*n* = 257), 55.3% (*n* = 249), and 40.2% (*n* = 181) of participants were afraid of what the police or criminal justice system, other government agencies, and healthcare professionals might think about their treatment. This study highlights those patients treated with CBMPs experience a high prevalence of perceived stigma from many corners of society. Future work should be undertaken to explore strategies to reduce perceived stigma at an individual and community level to avoid discrimination of patients, likely increasing appropriate access.

## 1. Introduction

A change in the legal status of cannabis-based medicinal products (CBMPs) in November 2018 allowed for doctors on the specialist register of the general medicinal council (GMC) to prescribe CBMPs for therapeutic use if clinically appropriate [1,2]. However, to this date prescription of CBMPs in the UK has been limited, with only small number of UK National Health Service (NHS) prescriptions being issued [2]. Despite the change in regulations there are still barriers to the integration of CBMPs in the UK [1]. These include rigid guidelines for prescribing CBMPs, a paucity of empirical evidence on the effectiveness of CBMPs, lack of education on how to safely prescribe CBMPs, and associated stigma of cannabis use in the UK [2,3,4]. It is well documented that stigma can reduce utilisation of healthcare services and can negatively impact treatment [5,6]. It can also lead to chronic stress and anxiety, in addition to subsequent mental and physical problems that can cause individuals to feel isolated and withdrawn [5,7], therefore limiting access [8]. Whilst there is a growing body of evidence on the associated effects of CBMPs on health-related quality of life in several health conditions [9,10,11], there is a paucity of knowledge on the prevalence and subsequent effects of stigma on the current and prospective UK patients [4].

Evidence from countries which have greater experience of CBMP therapies show stigma to be a factor in both prescribing practice and patient perception; in particular, perceived stigma from healthcare officials and society more broadly [4,12,13]. Semi-structured interviews of Canadian patients treated with CBMPs have highlighted reports of perceived stigma, especially from health care providers, law enforcement, and close relatives [12]. Participants in this study reported being especially affected by perceived stigma from healthcare providers [12]. Some participants had been inappropriately labelled as addicts, whilst others were incorrectly assumed to be accessing the medication for reasons other than legitimate health conditions [12]. Consequently, some patients did not disclose their medication use to healthcare providers, placing themselves at risk of drug–drug interactions [12]. Another qualitative study conducted in California illustrated similar effects of stigma on patients treated with CBMPs [13]. This often led to delays in seeking treatment or attempts to bypass their normal medical team [13]. In a study of 984 members of medical cannabis dispensaries, in northern USA, participants reported worries of being labelled as a “pothead” or “stoner”, and due to this perceived stigma, they reported seeking medical cannabis from healthcare providers that they did not have a long-term relationship with [5]. These findings suggest that there is ongoing stigmatisation of patients using CBMPs and this can have detrimental effects on effective implementation of CBMPs with respect to both access and safety.

Whilst the literature suggests ongoing stigmatisation of patients using CBMPs in mature markets, there is a paucity of research investigating the perceived stigma attached to patients with CBMP prescriptions in the UK. Therefore, it is difficult to establish the prevalence of stigma and the effect it could have on the integration of CBMPs. A recent review by Schlag et al. suggested that the use of CBMPs is still continually stigmatised based upon their association with recreational cannabis which is illegal in the UK [4]. In addition to removing other barriers to access, it is important to identify and address stigma in parallel to ensure that the full potential of CBMPs is realised.

Whilst it has been hypothesised that stigma is implicated in the challenges in accessing CBMPs in the UK, the extent of its prevalence is not yet known. The current study therefore aimed to investigate the perceived stigma attached to patients prescribed CBMPs across a variety of domains and agencies, including healthcare settings to establish its prevalence in a UK population. The results will provide a baseline for future research as well as highlighting the UK agencies and domains where perceived stigma toward medical cannabis use is the most prevalent. This will allow future research to explore accurate and tailored strategies to reduce the stigma toward patients treated with CBMPs to avoid further discrimination and allowing for appropriate access.

## 2. Methods

### 2.1. Participants

In total, 2319 patients actively treated with CBMPs for any indication at Sapphire Medical Clinics were invited to take part in this cross-sectional questionnaire study. Sapphire Medical Clinics is the first clinic providing treatment with CBMPs to be registered with the UK healthcare regulatory agency, the Care Quality Commission. Participants were invited via an email from the clinic to participate in the study. They were directed to an online link to the survey which was delivered via Qualtrics (Seattle, Washington, United States). Participants provided informed consent prior to completion of the questionnaire. Ethical approval for the study was granted by The University of the West of Scotland School of Education and Social Sciences Ethics committee approval number: 16648-13930.

### 2.2. Materials

A questionnaire was developed for dissemination via an electronic form to establish patient demographics, in addition to perceived stigma. This was constructed utilising a multidisciplinary group of clinicians and academics from the fields of psychology, neuroscience, and clinical medicine. This process was informed by literature review of previously reported experience of patients [14]. The design of the questionnaire was carefully considered to minimise the cognitive burden on patients, but also maximise the richness of information to be gained from the questionnaire. Pilot testing was performed to assess content validity and feasibility was confirmed through pilot testing by the Sapphire Medical Clinics Patient and Public Involvement Group (*n* = 7). Patients participating in this pilot study allowed for the questions to be refined and informed alterations to the questionnaire to improve clarity and structure.

### 2.3. Data Treatment

Data presented were either reported as descriptive statistics or the raw responses from participants, Results were presented as mean ± standard deviation [SD]) or median (interquartile range [IQR]) depending on whether the data was parametric or non-parametric. A one-way repeated measures ANOVA was performed to compare the perceived stigma from different segments of society. Statistical significance was defined as *p* < 0.050. A post-hoc analysis with Fischer’s least significant difference test was performed to compare individual variables if the ANOVA was statistically significant. All data analysis was performed using IBM Corp. Released 2017. IBM SPSS Statistics for Windows, Version 25.0. Armonk, NY, USA: IBM Corp.

## 3. Results

A total of 631 patients responded to the survey from 2319 patients initially invited to participate, 450 patients with complete responses (19.4% of the total number of patients receiving CBMPs invited to take part). Of those participants, 176 (39.1%) identified as female and 258 (57.3%) identified as male. The mean age they started consuming cannabis was 28.0 (±16.3) years old.

**Figure 1:**
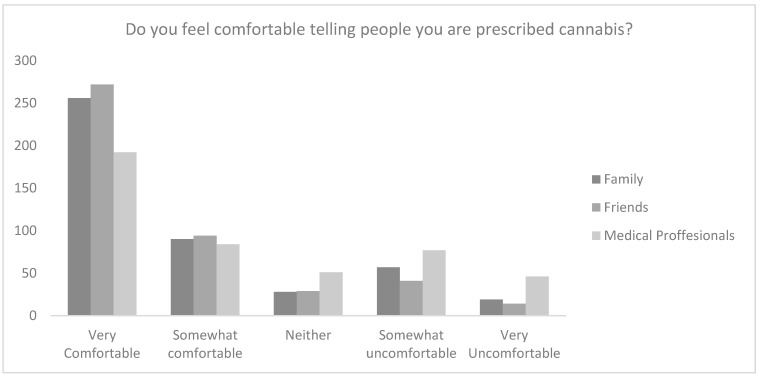
Raw responses to the question “Do you feel comfortable telling people you are prescribed cannabis?”.

Overall, 84.4% (*n* = 380) of participants believed that those who receive treatment with CBMPs are subject to stigma. Participants, however, were comfortable speaking about their prescription, with 81.3% (*n* = 366) participants responses reporting feeling very comfortable or comfortable telling friends, 76.9% (*n* = 346) telling family, and 61.3% (*n* = 276) telling medical professionals (Figure 1).

When applying a 5-point Likert scale there was a statistically significant difference between comfort with telling family (4.13 ± 1.22), friends (4.26 ± 1.11) and medical professionals (3.66 ± 1.42; *p* < 0.001). Post-hoc analysis revealed that the statistical difference was between medical professionals and both friends and family (*p* < 0.001).

**Figure 2:**
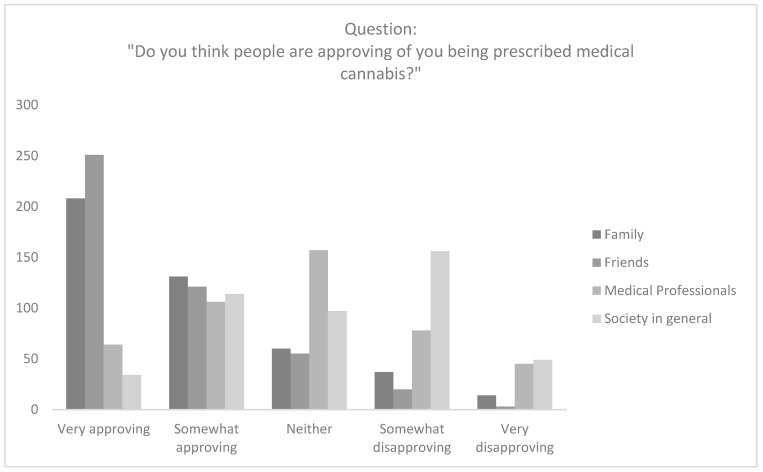
Raw responses to the question “Do you think people are approving of you being prescribed medical cannabis?”.

Participants largely thought that friends (*n* = 372; 82.7%) and family (*n* = 339; 75.3%) were very approving or somewhat approving of their CBMP prescription. However, participants thought that only 37.8% (*n* = 170) of healthcare professionals and 32.9% (*n* = 148) of society in general were very approving or somewhat approving of their CBMP prescription (Figure 2).

There was a significant difference between perceived approval from family (4.07 ± 1.09), friends (4.33 ± 0.92), medical professionals (3.66 ± 1.42) and society more broadly (2.84 ± 1.14; *p* < 0.001). Post-hoc analysis revealed that the statistical difference was between medical professionals and both friends and family (*p* < 0.001). Post-hoc analysis with a Fisher’s least significant difference test revealed that in comparison to family and friends, patients believed medical professionals (*p* < 0.001) and society (*p* < 0.001) were less approving of them being prescribed medical cannabis.

**Figure 3:**
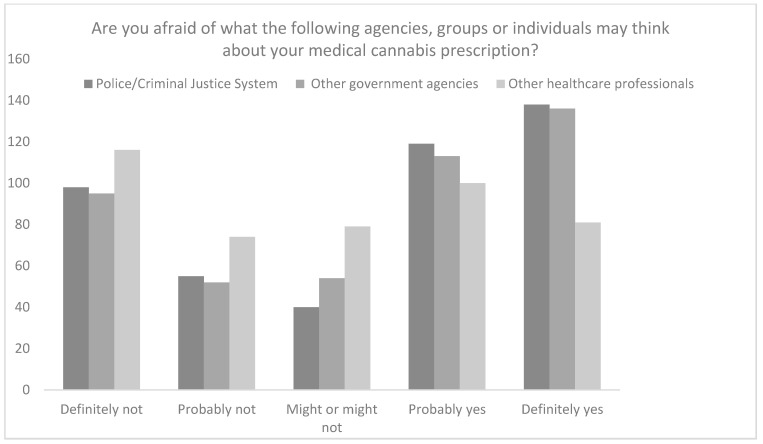
Raw responses to the question “Are you afraid of what the following agencies, groups or individuals may think about your medical cannabis prescription?”.

However, when asked about CBMPs and what the police or criminal justice system, other government agencies, and healthcare professionals, respectively might think about them receiving treatment with CBMPs 57.1% (*n* = 257), 55.3% (*n* = 249), and 40.2% (*n* = 181) of participants were concerned about how they might be perceived by each system, respectively (Figure 3).

On application of the 5-point Likert scale, there was a difference in perceived stigma from the police and criminal justice system (3.32 ± 1.54), other government agencies (3.32 ± 1.52) and other health professionals (2.90 ± 1.46; *p* < 0.001). Participants were more concerned with the police/criminal justice system and other government agencies being aware that they are a medical cannabis patient compared to health professionals (*p* < 0.001). However, on post-hoc analysis there was no difference between perceived perception from the police/criminal justice system and other government agencies (*p* > 0.050).

## 4. Discussion

Patients being treated with CBMPs were found to be comfortable discussing their treatment with family and friends. However, they were less comfortable discussing their prescription with health professionals. Moreover, there was a clear indication that there was perceived stigma by those being treated with CBMPs. Perceived stigma comes from society in general, with an emphasis on government agencies, even though it has been legal to access CBMPs since November 2018.

The data from this study support previous findings for other jurisdictions indicating that despite their legal status CBMPs, or more accurately the individuals being prescribed them, feel stigmatised [4,5,12,13]. It should be noted however that the USA and Canada, where a significant amount of prior data comes from, CBMPs exist alongside legal recreational cannabis markets. It has been observed that the co-existence of route to access CBMPs alongside recreational cannabis is a factor that increases stigmatisation [4,12,13]. However, our data reflect a similar pattern of perceived stigma despite there not being a legal recreational market for cannabis in the UK. Interestingly, when comparing the present data with that from researchers in California where cannabis for therapeutic purposes has been legal since 1995 [13], there is still significant concern from patients who are unwilling to seek out or discuss a legal therapeutic prescription of CBMPs. This suggests that additional strategies for education and awareness are necessary to address stigma, rather than being reliant upon changing attitudes over time.

The implications of stigma towards CBMPs are potentially barriers to seeking treatment in the UK. As previously noted, this is disappointing considering the unmet clinical need of refractory chronic illness, such as pain and anxiety, which may otherwise benefit from a trial of CBMPs [11,15]. Compared with previous research [4,5,12,13] this study shows that patients report feeling uncomfortable in seeking treatment despite its legal status. Patients appear to report being happy to discuss their treatment with friends and family, however, do not report being as comfortable when discussing CBMPs with medical professionals and other government agencies. Our data show that there is significant concern in relation to the criminal justice system despite the legality of their prescription. This creates an important profile of patients views on being prescribe CBMPs which warrants further investigation, especially in how this might affect stress and anxiety levels, when being prescribed CBMPs.

There is a notable lack of literature addressing the issue of stigma in patients using CBMPs, particularly in the UK where this is the first study to seek to answer this question. The present study, however, is limited in scope and is only able to evaluate the incidence of perceived stigma and is not able to explain why this stigma exists and how it changes patient behaviour. However, assessing whether perceived stigma exists is an integral first step in addressing its impact. Further qualitative research through semi-structured interviews or focus groups with UK patients and the general public may help to uncover these gaps in knowledge and illuminate how to best address stigma through education and research. The present study is also limited to respondents from one clinic in the UK. However, Sapphire Medical Clinics were the first clinic registered with appropriate regulators and have set up the UK Medical Cannabis Registry, which is the largest prospective observational cohort dataset of clinical outcomes following CBPM therapy of its kind in Europe [9,10,11]. Moreover, they treat patients across all four nations in the UK and Channel Islands, ensuring geographic diversity.

## 5. Conclusions

This study highlights that there is a high prevalence of perceived stigma towards patients treated with CBMPs from society, government officials, medical professionals, and the criminal justice system. Reduction in perceived stigma would likely increase appropriate access to CBMPs, as well as providing auxiliary benefits. Importantly, it would improve the safety of CBMPs with patients being empowered to share their full medication history with healthcare professionals. Future work should be undertaken to explore why this stigma exists in UK society, how it affects patient behaviour, and strategies to reduce stigma at an individual and community level to avoid discrimination of patients, such as education initiatives.

## Data Availability

Data are available upon request to the corresponding author.
